# Long-lived metastable knots in polyampholyte chains

**DOI:** 10.1371/journal.pone.0287200

**Published:** 2023-06-14

**Authors:** Masoumeh Ozmaian, Dmitrii E. Makarov

**Affiliations:** 1 College of Engineering, West Texas A&M University, Canyon, Texas, United States of America; 2 Department of Chemistry, University of Texas at Austin, Austin, Texas, United States of America; 3 Oden Institute for Computational Engineering and Sciences, University of Texas at Austin, Austin, Texas, United States of America; State University of New York at Binghamton: Binghamton University, UNITED STATES

## Abstract

Knots in proteins and DNA are known to have significant effect on their equilibrium and dynamic properties as well as on their function. While knot dynamics and thermodynamics in electrically neutral and uniformly charged polymer chains are relatively well understood, proteins are generally polyampholytes, with varied charge distributions along their backbones. Here we use simulations of knotted polymer chains to show that variation in the charge distribution on a polyampholyte chain with zero net charge leads to significant variation in the resulting knot dynamics, with some charge distributions resulting in long-lived metastable knots that escape the (open-ended) chain on a timescale that is much longer than that for knots in electrically neutral chains. The knot dynamics in such systems can be described, quantitatively, using a simple one-dimensional model where the knot undergoes biased Brownian motion along a “reaction coordinate”, equal to the knot size, in the presence of a potential of mean force. In this picture, long-lived knots result from charge sequences that create large electrostatic barriers to knot escape. This model allows us to predict knot lifetimes even when those times are not directly accessible by simulations.

## 1. Introduction

Just like a long piece of cord tends to be entangled causing nuisance, long polymers, particularly biopolymers, can be knotted, often with significant biological consequences. Indeed, knotted DNA were first observed in 1976 [1]; DNA knots have the ability to wreak havoc on replication, and cell machinery exists specifically with the purpose to “undo” the knots [2]. Knots in proteins have been discovered 24 years later [3] and, recently, systematic computer-aided searches through the protein databank have revealed many knotted proteins [4–6]. In parallel, experimental single-molecule studies have revealed knot dynamics at atomistic scales [7, 8].

The role played by knotted biopolymers in living systems is far from being understood. In addition to hampering replication or weakening polymer strands mechanically [9], it has also been speculated that knots slow protein degradation by the proteasome [10, 11]. More generally biopolymer translocation through biological pores, a key process in biology, is affected by the presence of knots [12–15]. Likewise, folding mechanisms of knotted proteins presents a significant theoretical challenge [16–22].

While a knot in a folded protein can exist as long as the protein remains folded, knots in disordered polymers tend to be transient. Yet an intriguing study [23] finds that a knot can survive in the denatured state of a protein over a very long time. Such knot stabilization may, in part, result from entropic effects, which render knots in very long polymer chains metastable as a result of knot self-tightening [24–26]. But given the relatively modest typical length of polypeptide chains, this purely entropic effect is unlikely to account for long-lived knots in unstructured polypeptides. Another possible explanation is that the steric hindrance that is due to side chains induces metastability of knots–this effect was studied in ref. 14 [27]. And yet another explanation is that, since proteins often have non-uniform charge distributions, intrachain electrostatic interactions may trap knots. Electrostatic interactions, indeed, have a significant effect on the thermodynamics of knotted charged polymers [28–30], but most of the studies of this effect have been limited to the case of uniformly charged polyelectrolytes such as DNA. Of course, some combination of the above three explanations may be required to account for the experimental observations.

**Fig 1:**
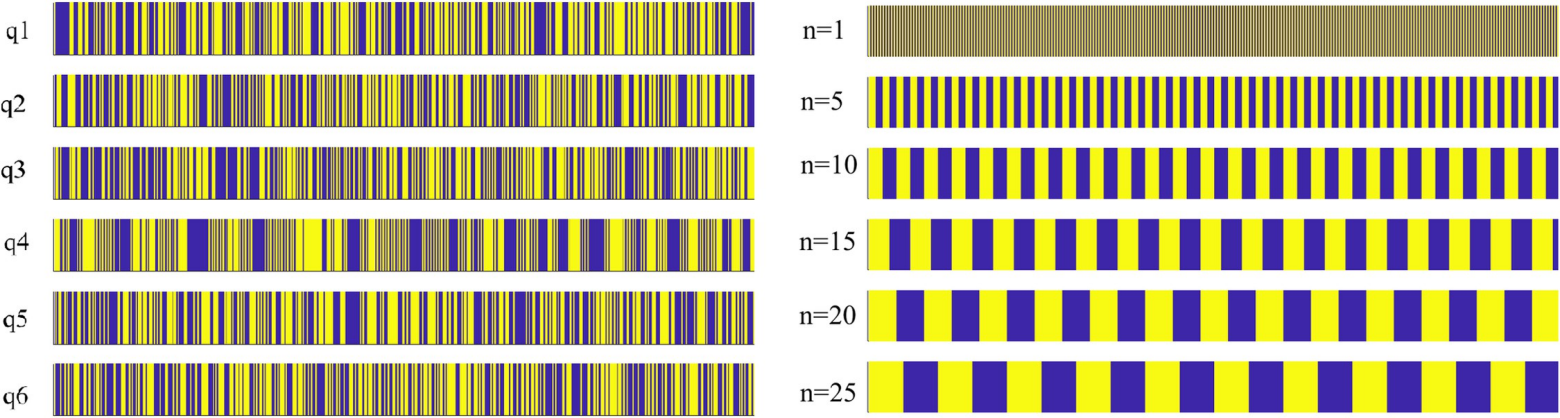
Charge sequences studied here. Left: “randomly” charged chains, with sequences labeled q1 to q6. Right: chains with diblock charge distributions, with same-charge block lengths *n* ranging from 1 to 25. Blue and yellow color stripes represent positively and negatively charged segments.

The purpose of this study is to explore the hypothesis that the polyampholyte nature of polypeptides (i.e., the fact that they tend to carry both positive and negative charges, while often been nearly electrically neutral) lengthen knot lifetimes. To this end, we have used coarse-grained Langevin dynamics simulations of charged polymer chains with varied charge distributions. Specifically, we consider several randomly generated charge sequences as well as diblock charge distributions with blocks of varied length (Fig 1). The “random” sequences considered here consist of monomers that carry charges ±1 selected with equal probabilities and with no correlation between monomer charges; of many possible such randomly generated sequences only a handful of those with a total charge of 0 or ±1 were selected. The sequences studied here are only a tiny subset of all possible sequences with these properties–a more extensive sampling of the sequence space would be very difficult here given the computational limitations.

**Fig 2:**
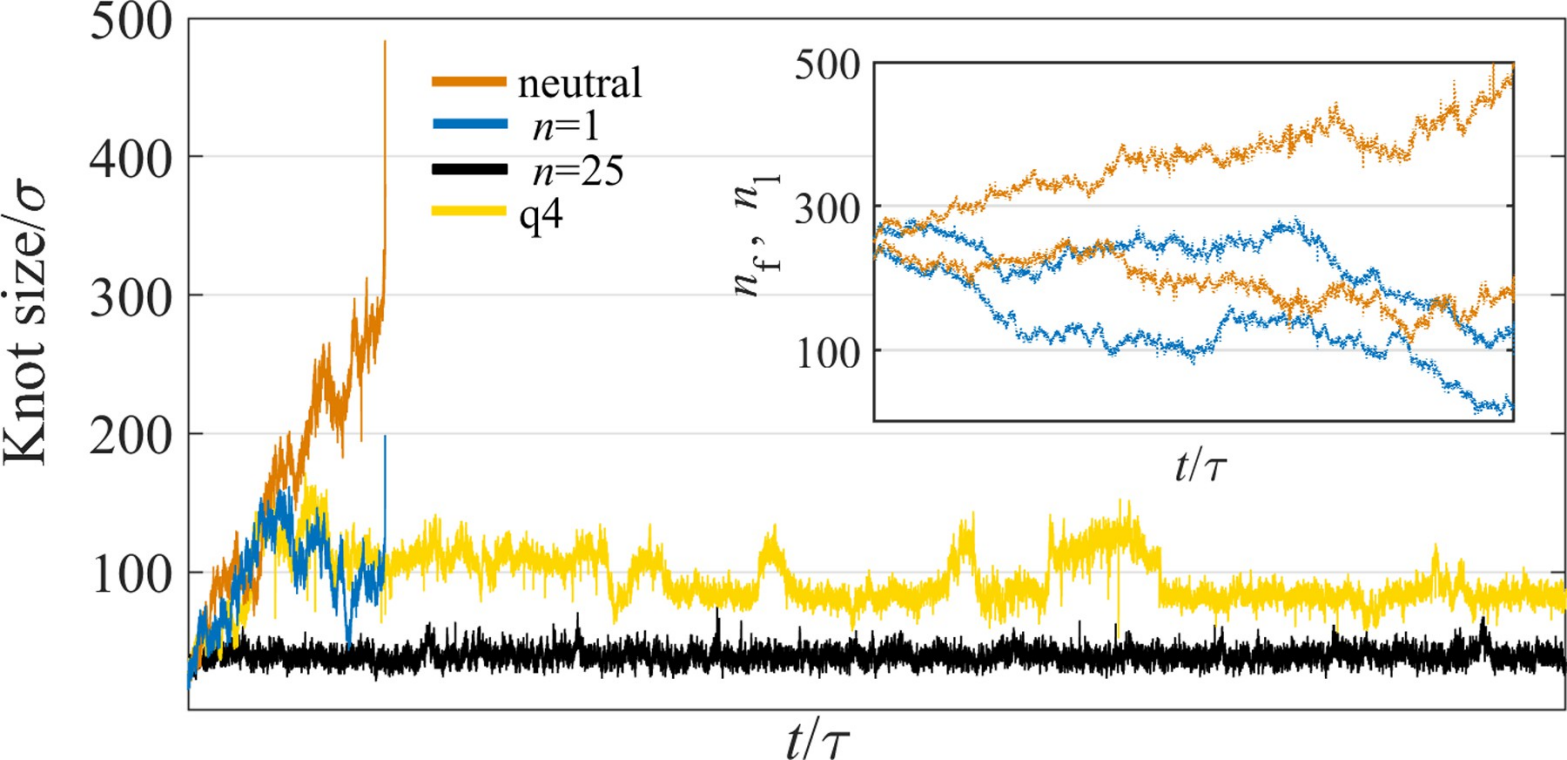
Simulated time dependence of the knot size (defined in the methods section) for an electrically neutral polymer chain, chains with diblock charge distributions (with block lengths *n* = 1 and 25) and one of the randomly charged polyampholyte chains (q4 sequence from Fig 1). The knots in the polyampholyte chain and in the diblock copolymer with *n* = 25 remain intact over the entire simulation time, whereas the knots in the neutral chain and in the chain with alternating positively and negatively charged monomers (*n* = 1) grow in size or diffuse along the chain towards the ends until they untie. Inset: time evolution of the knot boundaries. Here *n*_*f*_ and *n*_*l*_ are the first and the last monomers participating in the knot, where the total chain length is *N* = 500. Data are shown for the neutral chain and for the chain with alternating charges. The unit of time *τ* is defined in Section 2.

**Fig 3:**
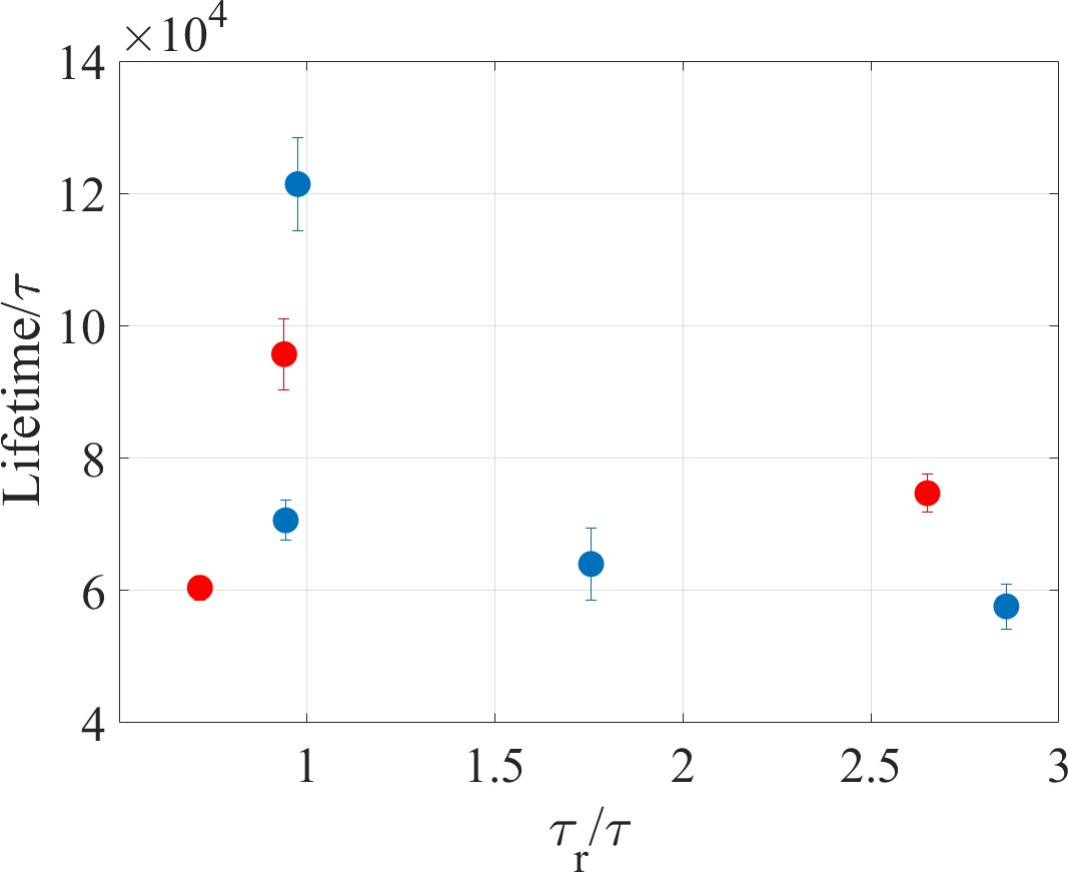
Relaxation times of the end-to-end distance of PA chains plotted against knot lifetimes for *n* = 1, 5, 10 and 15 diblock (blue circles) and for the q1, q2 and q3 charge sequences (red circles).

As illustrated in Fig 2, the knot dynamics and stability depend strongly on the charge distribution. In particular, while the trefoil (3_1_) knot in an uncharged chain is disentangled quickly, knots become metastable in some of the polyampholyte chains, exhibiting long lifetimes (sometimes exceeding the timescales directly accessible by our simulations). In particular, for polyampholyte chains with diblock charge distributions, knot lifetimes generally increase with the size *n* of blocks of the same charge. In contrast, we could not find any simple sequence-dependent parameter allowing one to predict the knot lifetime for random charge distributions (see SI.4 in S1 File). For instance, in electrically neutral homopolymer chains, knot lifetimes are known to be correlated with the global relaxation times of the chain [31, 32]. This is however not the case for the randomly charged polymers considered here. Indeed, consider the relaxation time of the chain defined as

$$\tau_{r}=\int_{0}^{\infty }ACF(t)dt$$

$$ACF(t)=\frac{<R(t)R(0)>-<R(0){>}^{2}}{<R(0)R(0)>-<R(0){>}^{2}}$$

where *ACF*(*t*) is the autocorrelation function of the end-to-end distance *R*(*t*). As seen from Fig 3, this time does not exhibit any clear correlation with the knot lifetime (see below for a description of how the knot lifetimes were computed).

Nevertheless, as will be shown below knot lifetimes in such randomly charged chains can be rationalized using a simple one-dimensional model, in which knot untying is viewed as progress along a one-dimensional reaction coordinate *x* in a potential of mean force *U*(*x*). Specifically, the knot size (defined below) provides a suitable reaction coordinate. The roughness of the potential *U*(*x*) caused by electrostatic interactions is then responsible for the slow untying dynamics.

The rest of this paper is organized as follows. Section 2 describes the simulation methods, Section 3 reports on the results, and Section 4 concludes with a discussion of the implications of this work.

## 2. Methods

### Simulation details

We model polymers as chains of charged beads connected by springs, with their dynamics obeying the Langevin equation. All beads have the same mass *m* and an effective diameter *σ*. All pairs of beads interact via a truncated Lennard-Jones (LJ) potential, which accounts for the excluded volume effects:

$$U_{LJ}(r)=\left\{\begin{array}{c} 4\varepsilon \left[{\left(\frac{\sigma }{r}\right)}^{12}-{\left(\frac{\sigma }{r}\right)}^{6}\right]+\varepsilon \;\;,\;\;\;\;r<r_{c}\\ \;\;0\;\;\;\;\;\;\;\;\;\;\;\;\;\;\;\;\;\;\;\;\;\;\;\;\;\;\;\;\;\;\;\;\;\;\;\;\;\;\;\;\;\;\;\;\;\;\;\;\;\;\;\;\;\;\;,\;\;\;\;r>r_{c} \end{array}\right.$$

Here *r* is the distance between the beads, *ε* is a strength of the LJ interaction and *r*_*c*_ = 2^1/6^σ is a cut-off distance. The parameter *ε* sets the energy scale of the system. Adjacent monomers are connected by a finitely extensible nonlinear elastic (FENE) spring potential given by

$$U_{FENE}(r)=-\frac{1}{2}k_{0}r_{0}^{2}\ln\left[1-{\left(\frac{r}{r_{0}}\right)}^{2}\right],$$

where *k*_0_ = 100(*ε* / *σ*^2^) is a spring constant, and *r*_0_ = 1.5σ is the bond’s maximum extension.

Any two charged monomers also interact via the Coulomb potential $U_{Coulomb}(r)=\frac{q_{i}q_{j}}{\varepsilon_{s}r}$, where *ε*_*s*_ is the dielectric constant of the solvent and *q*_*i*_ and *q*_*j*_ are the monomer charges. Each charged monomer carries a charge ±*e*. The electrostatic parameters of the system were chosen such that the Bjerrum length *l*_*B*_ = *e*^2^/(*ε*_*s*_*k*_*B*_*T*)is equal to the bond length *σ*.

The Langevin equation describing the dynamics of the monomers was integrated using the velocity Verlet algorithm [33] with a time step of *dt* = 0.002*τ*, where $\tau =\sigma \sqrt{m/\varepsilon }$ is the LJ unit of time. The temperature was set at *T* = *ε* / *k*_*B*_, where *k*_*B*_ is Boltzmann’s constant.

It should be noted that, while various types of knots, including figure-eight (4_1_), Gordian (5_2_) and stevedore (6_1_), have been identified within protein structures [19], here we choose to study the simplest, most common, and most studied trefoil knot (3_1_). Studying knots with more complicated topology can however also be of great interest.

### Observables and analysis

**Fig 4:**
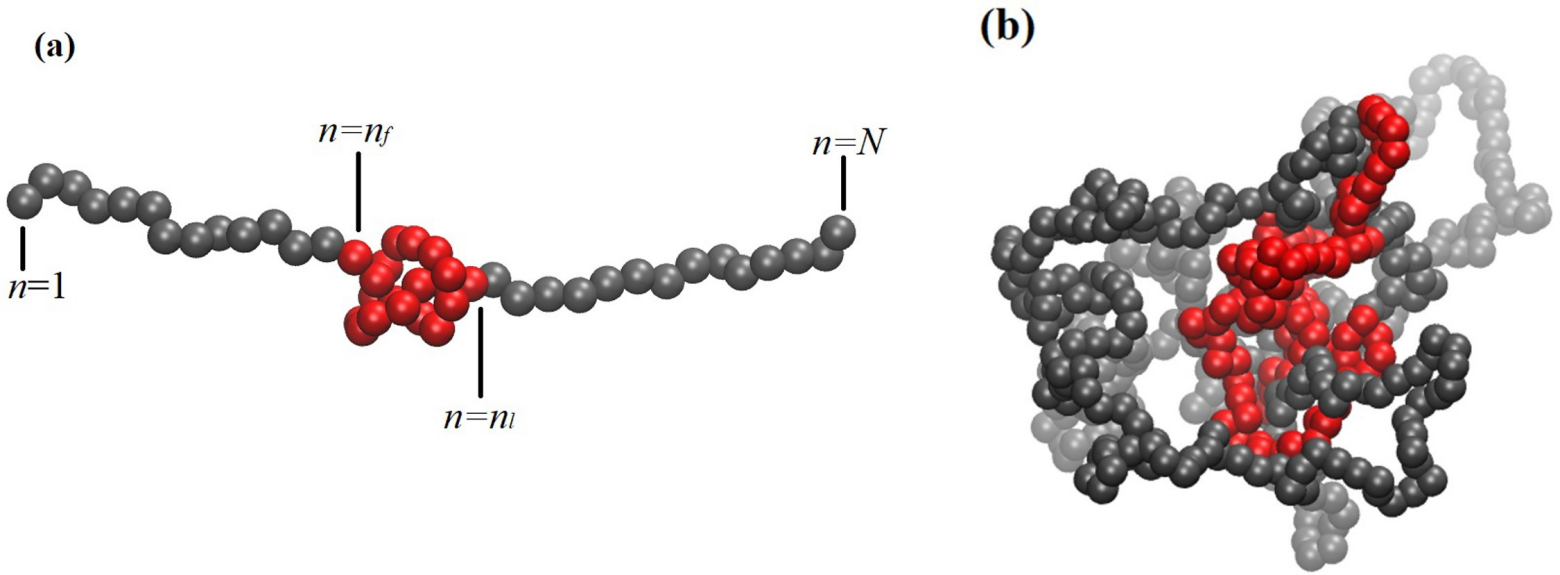
Knot in a polymer chain. Monomers in red represent the knot region. a) The knot size is defined as the number of monomers *n*_*f*_ -*n*_*l*_ in the knot region, and the knot position is defined as (*n*_*f*_ +*n*_*l*_)/2, where *n*_*f*_ and *n*_*l*_ are the first and the last monomers participating in the knot, with the monomers being numbered sequentially from one chain end (*n* = 1) to the other (*n* = *N*). b) A snapshot of a typical knotted chain configuration (here the sequence is q1 after simulation begins. PA chains tend to collapse to dense structures as a result of electrostatic interactions.

Knot trajectories were analyzed frame by frame using the KymoKnot software package [34–36] for linear chains, which uses the Alexander polynomial, a knot invariant, to determine the topology of the knot [37]. We use the analysis data produced by the bottom-up method in KymoKnot, which is a more accurate choice for our compact knotted chains [35] (Fig 4B). The two main observables extracted from this analysis are the knot size and the knot position along the chain (Fig 4A). Let *n*_*f*_ and *n*_*l*_ are the first and the last monomers participating in the knot, with the monomers being numbered sequentially from one chain end (*n* = 1) to the other (*n* = *N*). Then the knot size is defined as the number of monomers *n*_*l*_ − *n*_*f*_ in the knot region, and the knot position is (*n*_*f*_ +*n*_*l*_)/2,

## 3. Results and discussion

### Knot lifetimes

The initial knotted chain configuration was created by placing a trefoil knot in the middle of the chain; the knot was tightened by applying opposing pulling forces of 2ε/σ. Starting with such an initial configuration, the dynamics of the chain was followed until the knot disappeared. For each charge distribution, the reported mean knot lifetime is an average over 40 independent trajectories. The measured mean lifetimes are shown on the horizontal axis of Fig 4, indicating that some charge sequences result in longer-lived knots than others. This effect cannot be explained by entropic stabilization, as all of the chains have the same length (*N* = 500), and an electrically neutral chain of this length is disentangled by thermal fluctuations rapidly (Fig 2). (See SI. 1 in S1 File for more information).

**Fig 5:**
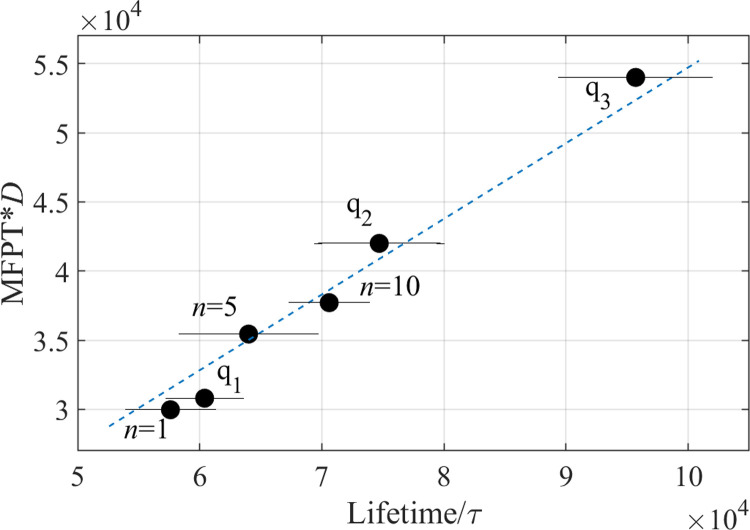
The product of the mean first passage time (*MPFT*) and the diffusion coefficient *D* estimated using Eq (1) and plotted against the mean knot lifetime obtained from simulations. The slope of the linear fit (dashed blue line) gives an estimate for the diffusion coefficient, *D* = 0.6 *σ*^2^ / *τ*, for the reaction coordinate *x* equal to the knot size.

As seen in Fig 5, the average knot lifetime in chains with diblock charge sequences increases with the block length *n*. In contrast, the average lifetime varies considerably among random charge sequences (we note that the parameter q1, q2, q3 here simply labels random sequences in ascending order–thus the apparent “correlation” between this parameter and the knot lifetime is a property of labeling and is not physically significant). In fact, for some of the sequences that are not included in Fig 5, knot lifetimes exceeded the simulation time and thus could not be measured directly (also see Fig 1). They could, however, be estimated using the model described and validated below.

### One-dimensional model of knot dynamics

While disentangling of a knot involves many degrees of freedom, several studies [38–40] have shown the utility of low-dimensional models in which the motion of the knot is viewed as one-dimensional diffusion along an appropriately chosen coordinate *x*, in the presence of an effective potential *U*(*x*). Intuitively, the knot may become disentangled either through its diffusion along the chain until it reaches its end [12, 40, 41], or as a result of increase in its size, or possibly via some combination of these two mechanisms. This suggests two plausible candidates for the coordinate *x*, the knot size and the position of the knot along the chain. The size growth mechanism turns out to be dominant in our case (see SI.2 in S1 File), and thus we use the knot size as the coordinate *x* measuring the progress of knot untying. (Note, however, that knot diffusion may be the dominant mechanism of untying for longer chains–in such cases the knot size may not be the appropriate reaction coordinate). We assume that the motion along *x* is governed by the overdamped Langevin equation

$$\frac{k_{B}T}{D}\dot{x}=-U^{\prime }(x)+f(t),$$

where *D* is a diffusion coefficient and *f*(*t*) is the delta-correlated Gaussian noise, whose strength is related to the temperature *T* and the diffusion coefficient via the fluctuation-dissipation theorem $\langle f(t)f(t^{\prime })\rangle =2D^{-1}{\left(k_{B}T\right)}^{2}\delta \left(t-t^{\prime }\right).$

**Fig 6:**
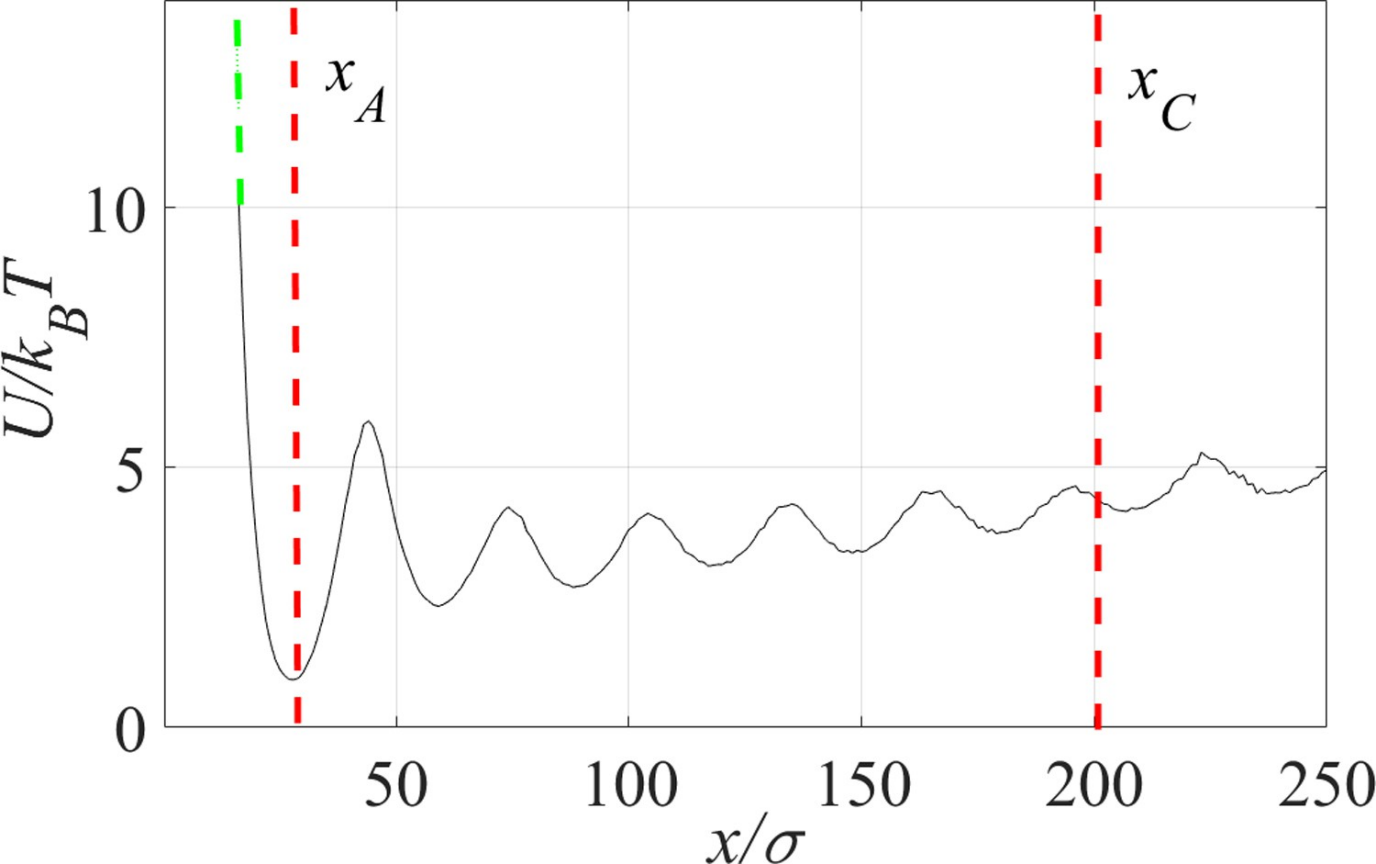
Potential of mean force as a function of the knot size for a polymer chain with a diblock charge distribution (*n* = 20). As *x* → 0 this potential, of course, must diverge preventing the knot from shrinking to zero size, but this high-energy region is not sampled by the simulation. Thus, for the purpose of evaluating the mean first passage time using Eq 1, the computed potential was extrapolated toward smaller values of *x* (i.e. tighter knots) such that it diverges for x→0 (green dashed line). The initial knot size is *x*_*A*_; the knot is considered untied when the coordinate *x* reaches a value *x*_*C*_−both of these values are indicated as vertical red dashed lines.

The definition of the effective potential *U*(*x*) requires some care. For an equilibrium system, it would be the potential of mean force, *U*(*x*) = −*k*_*B*_*T* ln *p*(*x*), where *p*(*x*) is the equilibrium distribution of *x* (i.e., of the knot size). But given that the knot is free to escape, most equilibrium chain configurations are unknotted, and thus the value *x* is not even defined for them. Our operational definition of *U*(*x*) is the potential of mean force in a modified system with a repulsive potential preventing knot escape, a scenario where the knot size distribution *p*(*x*) is well defined. The potential of mean force is then computed directly from the observed probability distribution *p*(*x*). We run the simulations until the observed values of *U*(*x*) are converged to within 10^−2^*k*_*B*_*T*—the small fluctuations observed in the PMF plot (Fig 6) are indicative of the remaining statistical errors. See SI. 3 in S1 File for details and further discussion. An example of the potential thus computed is shown in Fig 6.

Assuming that the initial knot size is *x* = *x*_*A*_ and that the knot is considered untied when it reaches a value *x*_*C*_, the knot lifetime within this model is the mean first passage time from *x*_*A*_ to *x*_*C*_, which is given by (see, e.g., ref. [42])

$$MFPT(x_{A}\to x_{C})=D^{-1}\int_{x_{A}}^{x_{C}}dye^{\frac{U(y)}{k_{B}T}}\int_{-\infty }^{y}dz\;e^{-\frac{U(z)}{k_{B}T}}$$ (1)

The choice of the boundaries *x*_*A*_ and *x*_*C*_ is illustrated in Fig 6. Note that Eq 1 in general depends on the values of the potential *U*(*x*) for *x* < *x*_*A*_ and, formally, even for *x* < 0, but, clearly *U*(*x*) must diverge for *x* → 0, as the knot cannot shrink to zero size or have negative size. Since high values of the potential corresponding to small knot sizes are not sampled by the simulations, we have extrapolated *U*(*x*) to smaller values of *x* as shown in Fig 5, green dashed line. The value of *x*_*A*_ is chosen to be the the potential minimum.

Within the one-dimensional diffusion model, long knot lifetimes or long mean first passage times result from high barriers that the system must overcome when escaping from the initial potential well. Indeed, a barrier significantly exceeding the thermal energy is observed in Fig 6, explaining the relatively long lifetime of the knot in a polymer with alternating sequences of *n* = 20 opposite and 20 negative charges.

More generally, the product *D* * *MPFT*(*x*_*A*_ → *x*_*C*_) calculated using Eq (1) with the computed potential of mean force *U*(*x*), is proportional to the mean knot lifetime *τ* measured directly (Fig 5), both validating the present one-dimensional model of knot disentanglement and allowing us to estimate the value of the diffusion coefficient in this model. This resulting value of *D* = 0.6σ^2^ / *τ* is somewhat unexpectedly high. Indeed, this value is comparable to the monomer diffusion coefficient of 1σ^2^ / *τ* suggesting that local chain motion, as opposed to global chain rearrangement, is responsible for the dynamics of untying. This result is consistent with the self-reptation and local breathing picture proposed earlier [38] as well as with fast diffusion of knot size observed in another simulation study of a circular, uniformly charged knotted chain [29].

Equipped with a low-dimensional description, we can now estimate knot lifetimes in polymers for which it was too long to be estimated directly. The knot lifetimes thus predicted, along with the knot lifetimes of the charge sequences which were estimated directly, are shown in Table 1.

**Table 1:** Mean knot lifetimes in ascending order for different charge distributions. The second column shows the lifetimes that are short enough to be measured directly in simulations. Lifetimes in the third column are predictions of Eq 1 for those sequences for which the mean knot lifetime was too long to be measured directly. We note that the parameter q1, q2, etc. simply labels the sequences and has no physical significance.

Charge sequence	Lifetime of the knot/10^4^τ (from simulation)	Lifetime of the knot/10^4^τ (from diffusion model)
***n* = 1**	5.7±0.3	5.5
**q1**	6.1±0.3	5.6
***n* = 5**	6.4±0.5	6.5
***n* = 10**	7.1±0.2	6.9
**q2**	7.5±0.5	7.7
**q3**	9.6±0.7	9.8
***n* = 15**	-	15.9
***n* = 20**	-	16.6
**q5**	-	22.4
***n* = 25**	-	39.1
**q6**	-	43.8
**q4**	-	45.2

## 4. Conclusions

In summary, while knots in intrinsically disordered polymer chains of modest length that are either electrically neutral or uniformly charged are short lived, we find that electrostatic interactions within a polyampholyte chain (i.e. a polymer that has both positive and negative charges, such as is the case for many proteins) may trap knots within a chain, significantly increasing the knot lifetime. This effect is due to the electrostatic interactions resulting in rough energy landscapes with barriers trapping the knots in metastable conformations. In combination with entropic effects [26] and steric hindrance [27, 43], this mechanism provides a possible explanation of the experimental observation of long-lived knots in denatured proteins [23].

For charge sequences with alternating blocks of positive and negative charges, the knot lifetime increases with the length of a block. For random charge sequences (subject to the net zero charge constraint) we have not been able to identify a simple sequence-based parameter that is a good predictor of the knot lifetime, with usual sequence-based measures [44, 45] used for intrinsically disordered proteins showing little correlation with the observed lifetime (SI. 4 in S1 File), although this conclusion should be viewed as tentative given the limited number of “random” sequences studied. Nevertheless, the dynamics of knots in such chains is well described as one-dimensional diffusion along a coordinate equal to the knot size, with the electrostatic interactions determining the effective potential acting along this coordinate. This model allows us to predict knot lifetimes for sequences where they cannot be measured directly.

## Supporting information

S1 File (DOCX)
