# Supplementary material for: Long-lived metastable knots in polyampholyte chains
Source: PLoS One. 2023 Jun 14;18(6):e0287200. doi: 10.1371/journal.pone.0287200 (PMC10266668; doi:10.1371/journal.pone.0287200)
Supplement: S1 File — (DOCX) [file pone.0287200.s001.docx]

**SI.1. Dependence of knot stability on chain length**

Motivated by earlier studies indicating that the knot lifetime strongly depends on the chain length *N* [1, 2], here we examine this dependence for uncharged chains and for a charged chain with (quasi)random charge sequences. In the latter case we start with the q4 charge sequence (*N*=500) and obtain its shorter fragments by successively removing 50-monomer fragments from both ends, resulting in shorter chains with N=400, 300, 200, and 100.


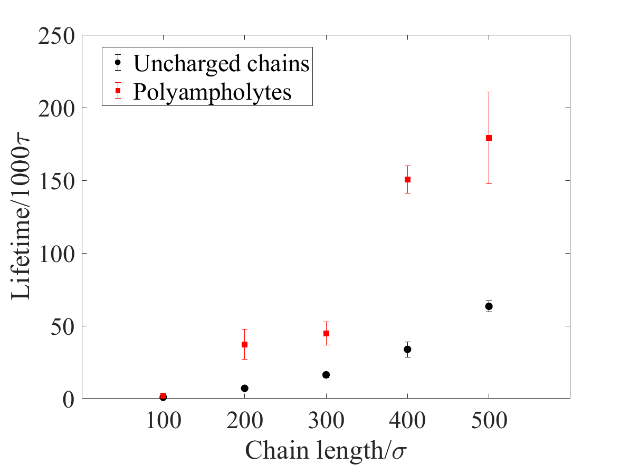


**Figure S1.** The knot lifetime grows with increasing chain length for both polyampholytes and uncharged chains. This lifetime is significantly greater for a polyampholyte chain as compared to the uncharged chain.

**SI.2. Knot size as the reaction coordinate**

Here we illustrate two possible “reaction mechanisms” for knot escaping a polymer chain, knot growth (Fig. S2, lower panel) and knot diffusion along the chain (Fig. S2, upper panel). In our simulations, only ~10% of untying events for each charge sequence occurred via knot diffusion, with knot growth thus being the dominant untying mechanism.

**
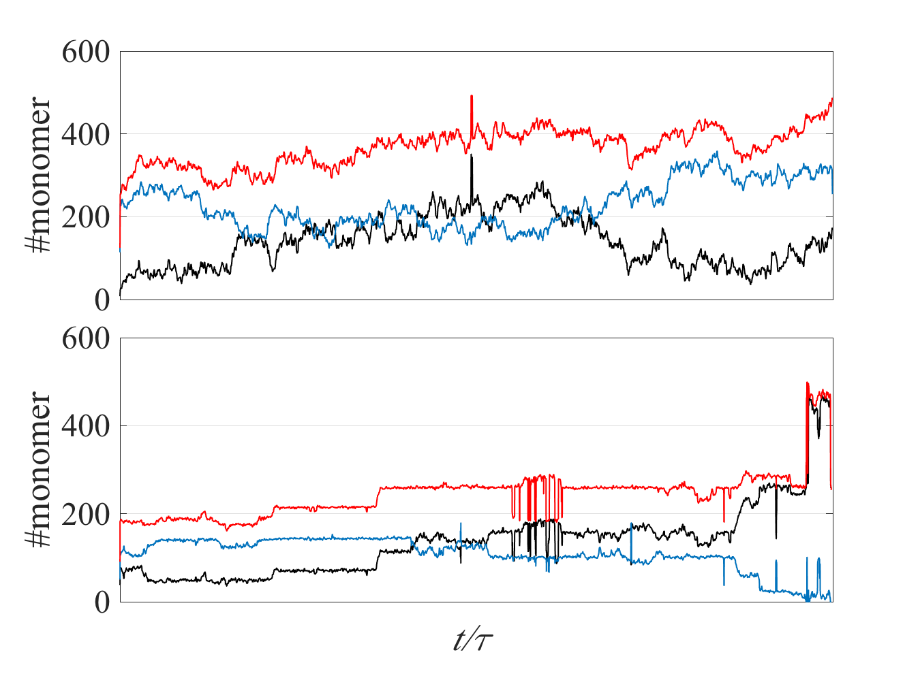
**

**Figure S2.** Knot size (black) and locations $n_{f},n_{l}$ of the first (blue) and the last (red) monomers on the knot region plotted as a function of time. In the upper panel, the knot diffuses along the chain until it escapes off one of its ends, with the knot size remaining smaller than 200 monomers. In the lower panel, the knot size grows until it becomes comparable to the chain length *N*~500, thereby completing the untying event.

**SI.3. Equilibrium properties of knots.**

While a knotted conformation of a polymer with free ends is, at best, a metastable rather than thermodynamically stable state, a knot can be trapped by closing the chain or by tethering its ends to repulsive walls that prevent the knot from escaping [3]. Here we use the second method, with the distance between the two walls was set to be twice the radius of gyration of the chain.

Figure S3 shows the computed potentials of mean force and, in particular, illustrates that sequences resulting in long knot lifetimes are characterized by rugged free energy landscapes with deeper wells.

| 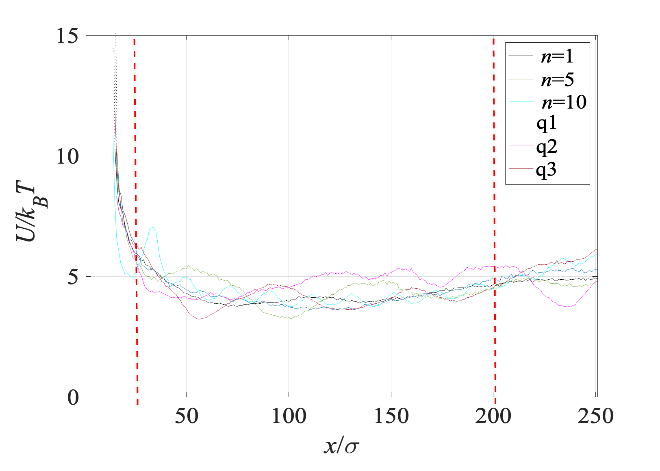  (a) | 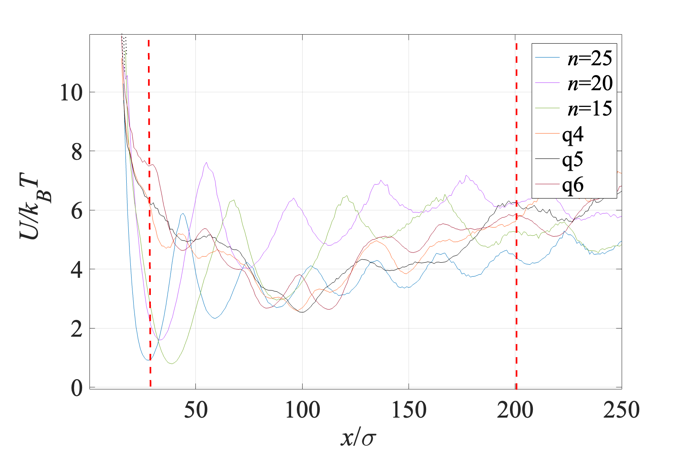  (b) |
| --- | --- |

**Figure S3.** (a) Potentials of mean force, $U\left( x \right), x$ being the knot size, for chains with short knot lifetimes (*n*=1, *n*=5, *n*=10, q1, q2, q3). (b) Potentials of mean force $U\left( x \right), x$ being the knot size, for chains with long knot lifetimes (*n*=15, *n*=20, *n*=25, q4, q5, q6). The dashed red lines represent the boundaries $x_{A}$ and $x_{C}$ used in the calculations of the mean first passage times (Eq. 1).

**SI.4. Lack of correlation of structural, and sequence chain parameters with the knot lifetimes.**

- - 1. **Chain compactness is uncorrelated with knot lifetime for random sequences.**

A long enough PA chain with a net charge smaller than a critical number (globally neutral charged chain) collapses to a dense globular state [4]. From a previous study of uncharged chains, we know that knots behave differently in the globules and in the coils [5]: the knot size is found to be small in the swollen phase and large in the dense phase, and compactness increases the likelihood of forming knots in free uncharged chains. Could knot lifetime also depend on the compactness of the chain? Figure S4 shows that chain compactness cannot account for the significant variation among chains with different charge sequences.

**
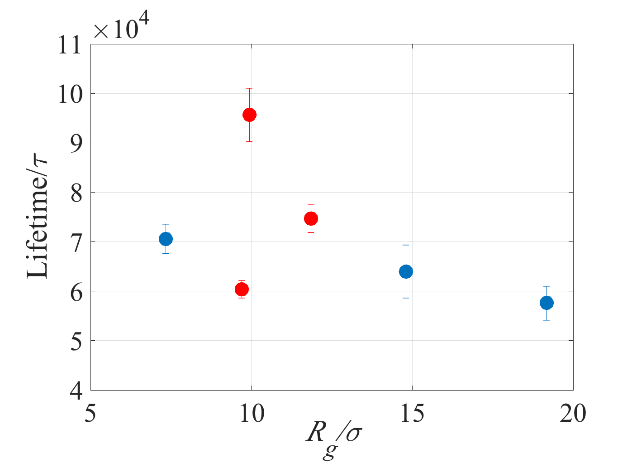
**

**Figure S4.** Knot lifetime is not significantly correlated with the radius of gyration of the PA chains, both for randomly charged sequences (red circles) and diblock charge sequences (blue circles).

- - 1. **Charge decoration metric does not predict the knot lifetime in PA chains with random sequences.**

Earlier studies have introduced different sequence-based metrics to quantify intra-chain electrostatic interactions and to predict conformational ensembles of intrinsically disordered proteins [6, 7]. For instance, Sawle and Ghosh [7] proposed a patterning parameter defined as where *N* is the total number of monomer/residues and *q_n_* is the charge carried by the monomer *m*. Figure S5 shows that such metric is not significantly correlated with the observed knot lifetime for random charge sequences


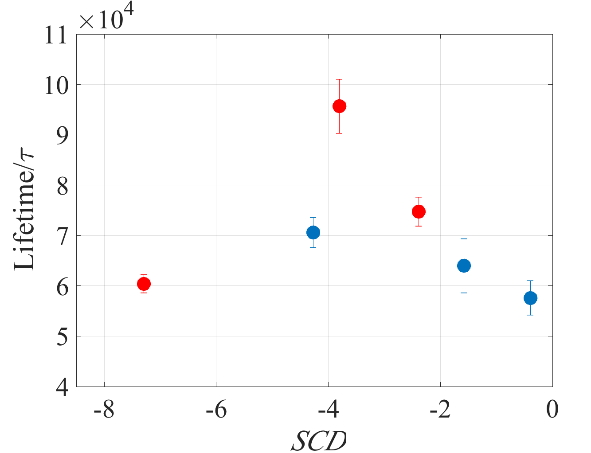


**Figure S5.** *SCD* calculated for PA chains with diblock (blue) and random (red) charge distributions plotted against the knot lifetime.

**References**

1 Metzler, R., et al., *Diffusion mechanisms of localised knots along a polymer.* EPL (Europhysics Letters), 2006. **76**(4): p. 696.

2. Grosberg, A.Y., *Critical exponents for random knots.* Physical review letters, 2000. **85**(18): p. 3858.

3. Dai, L. and P.S. Doyle, *Trapping a knot into tight conformations by intra-chain repulsions.* Polymers, 2017. **9**(2): p. 57.

4. Wittmer, J., A. Johner, and J. Joanny, *Random and alternating polyampholytes.* EPL (Europhysics Letters), 1993. **24**(4): p. 263.

5. Virnau, P., Y. Kantor, and M. Kardar, *Knots in globule and coil phases of a model polyethylene.* Journal of the American Chemical Society, 2005. **127**(43): p. 15102-15106.

6. Das, R.K. and R.V. Pappu, *Conformations of intrinsically disordered proteins are influenced by linear sequence distributions of oppositely charged residues.* Proc Natl Acad Sci U S A, 2013. **110**(33): p. 13392-7.

7. Sawle, L. and K. Ghosh, *A theoretical method to compute sequence dependent configurational properties in charged polymers and proteins.* The Journal of chemical physics, 2015. **143**(8): p. 08B615_1.
